# Inflammation Progresses to Normal Tissue in Patients with Anthracosis after Discontinuation of Exposure to Fossil Fuel

**DOI:** 10.2174/18743064-v16-e2203310

**Published:** 2022-05-31

**Authors:** Mohammad Samet, Fariba Binesh, Sanaz Zand, Mohammad Rezaeisadrabadi, Ryan Nazemian

**Affiliations:** 1 Department of Internal Medicine, Shahid Sadoughi Hospital, Shahid Sadoughi University of Medical Sciences, Yazd, Iran; 2 Department of Pathology, Shahid Sadoughi Hospital, Shahid Sadoughi University of Medical Sciences, Yazd, Iran; 3 School of Medicine, Shahid Sadoughi University of Medical Sciences, Yazd, Iran; 4 Department of Internal Medicine, Shahid Beheshti University of Medical Sciences, Tehran, Iran; 5 Institute for Transformative Molecular Medicine, Case Western Reserve University, 10900 Euclid Ave, Cleveland, OH 44106, United States

**Keywords:** Anthracosis, Bronchoscopy, Fossil fuel, Inflammation, Tuberculosis, Fibrosis

## Abstract

**Background:**

Exposure to toxic materials predisposes the lungs to infectious agents and inflammatory responses. The present study was performed on patients with anthracosis caused by exposure to fossil fuels in previous years, and histopathological features of airways’ normal-appearing tissue were compared with histopathological features of anthracotic plaques in these patients.

**Methods:**

Bronchoscopic evaluations were performed on bakery workers who were directly in contact with fossil fuels. Samples were taken from anthracotic plaques (Group A) or seemingly intact tissues at their periphery (Group B). Pathological evaluations were done after hematoxylin and eosin staining. Then, microbiological cultures were performed for the diagnosis of *Mycobacterium tuberculosis*. Data obtained from bronchoscopy, pathology, and cultures were compared between anthracotic and normal-appearing peripheral tissues using chi-square and analysis of variances (ANOVA) at a 95% confidence level.

**Results:**

Sixty-eight patients were diagnosed with anthracotic plaques. The mean ± SD of the patients’ age was 72.12 ± 13.74 years. Females comprised 58.8% of the sample, and 85.3% of the patients were Iranian. The frequency rates of disseminated plaques and obstructive types were 86.8% and 48.5%, respectively. Ten patients (14.70%) were diagnosed with tuberculosis, and 4.41% (3 of 68) had granuloma, which was detectable only in samples gathered from Group A. Fibrosis was more common in Group A (10.3%, *p* = 0.03), and most of the evaluated samples in both groups exhibited inflammatory features.

**Conclusion:**

Inflammatory changes and tissue damage can be seen in anthracotic plaques and the surrounding normal-appearing tissue, even after removing the triggering factors. So, it is suggested to take a biopsy from seemingly intact tissue at the periphery of the anthracotic plaque when a biopsy is needed in a patient with anthracosis to reduce the risk of bleeding. Besides, medical treatment should be done to control inflammation.

## 1 INTRODUCTION

The term ‘anthracosis’ was coined in 1813 by Pearson to refer to a condition characterized by the pigmentation of the lung tissue. It is considered an occupational disease mostly observed in coal miners [1]. While disease signs are primarily present in lung tissue, it also affects other organs such as the esophagus, liver, and spleen [2-4]. The prevalence of anthracosis is roughly estimated to be between 3.4% and 21%, based on data available on patients who had to undergo bronchoscopy for other causes of pulmonary diseases [2, 5]. Exposure to dust particles from different sources, including coal, tile, mica, silica, aluminum, and silicon, and biomass smoke, has been proposed to be involved in the etiology of anthracosis. Quartz and iron deposits are the most etiological plaques found in the bronchial lumen of patients suffering from anthracosis [6-8]. Studies have shown that tuberculosis (TB) is significantly more frequent in patients with anthracosis [8-14]. Anthracosis’s histopathologic features include anthracotic nodules with black pigmentation (carbon-like particles) in macrophage cytoplasm, reactive hyperplasia, and submucosal fibrosis. In TB-associated anthracosis, granulomas may also be seen [2, 15].

The main clinical manifestations of anthracosis include cough and dyspnea. During the physical examination of the lungs, patients will usually show wheezing, rales, or decreased breathing sounds on auscultation, even though some patients may have a normal physical examination. Furthermore, spirometry is unreliable in diagnosing the condition as it bears no association with the severity of clinical findings [8, 16-19]. Radiological assessment, especially a computerized tomography (CT) scan, is more accurate than other techniques for differential diagnosis of anthracosis, and it is so helpful to detect mediastinal or hilar lymphadenopathy with/without lymph node calcification, bronchial stenosis (unilateral or bilateral), atelectasis, and mass lesions [20-22]. However, the standard gold method for diagnosing anthracosis is bronchoscopy, which can clarify the presence of black discoloration in bronchi mucosa.

Biopsy sampling from the plaque mucosa is complex and commonly accompanied by bleeding [16, 23, 24]. Moreover, phenomena, such as bronchial narrowing, swelling with infiltration, or obstruction can be identified during bronchoscopy. Treatment of anthracosis includes conservative and anti-inflammatory medications. However, in the case of TB-associated anthracosis, anti-TB drugs are also prescribed. For cases of severe obstruction, bronchial stents may be used [2, 22, 23, 25].

In the bronchoscopic examination of anthracotic patients, normal-appearing tissues are usually not appropriately examined. Accordingly, biopsies from anthracotic plaques can be replaced by biopsies from normal-appearing tissues, thus reducing the risk of bleeding during bronchoscopy. In the current study, the histopathological features of biopsy samples taken from bronchial anthracotic plaques were compared with histopathological features of peripheral normal-appearing tissue. We hypothesized that there is no significant difference between biopsies taken from these two locations.

## 2 METHODS

### 2.1 Study Design and Participants

This cross-sectional study included the patients referred to Yazd Shahid Sadoughi Hospital from 2013 to 2016. Inclusion criteria included the retired bakery workers and the patients who contacted fossil fuels, but they have not contacted them for five years. To ensure that contact with fossil fuels is stopped, patients’ working and living conditions, especially heating devices, were questioned, and a 5-year period away from exposure was considered one of the inclusion criteria. Moreover, the bronchoscopy indications in these patients were respiratory problems such as chronic cough, dyspnea, and others, with a negative result of sputum smear and culture for TB and a negative CT scan for malignancy. Also, patients were excluded with active inflammatory, infectious disease, known tuberculosis, smoking history, or employment history in particular occupations such as digging wells and mining. The study protocol was approved by the ethics committee of the Shahid Sadoughi University of Medical Science, and written informed consent was obtained from all participants for using their physical examination, medical history, and hospital records in this study.

### 2.2 Bronchoscopy and Biopsy Sampling

We performed bronchoscopy (OLYMPUS, CV-260SL) for each patient, and six tissue biopsies were taken from two separate locations: 3 from anthracotic plaques (Group A) and three from seemingly intact tissues at the periphery of the lesions (Group B). They were put into two separate solutions containing formalin denomination and randomly labeled samples as groups 1 and 2. The samples underwent fixation, embedding, sectioning, and staining with hematoxylin and eosin. Then, they were given to an independent pathologist for evaluation who was not aware of randomization. Samples were also sent to the clinical laboratory to test tuberculosis according to the standard operating procedures for *Mycobacterium tuberculosis* (M. tb) detection. Furthermore, bronchoalveolar lavage (BAL) fluid from involved bronchi was collected for cytology study and *M. t*b smear and culture.

### 2.3 Study Variables

The study variables included patients demographics (age, gender, and nationality), bronchoscopic findings (bronchial obstruction and plaque spreading [localized or disseminated unilaterally/bilaterally]), histopathological features (presence of acute or chronic inflammation, fibrosis, granulomatosis, metaplasia, dysplasia, neoplastic cells), and the results of smear and culture tests on BAL fluid and bronchial tissues. Inflammation was categorized as mild (10-20 cells/hpf), moderate (20-50 cells/hpf) and severe (more than 50 inflammatory cells/hpf).

### 2.4 Data Analysis

The data were analyzed using SPSS statistical software (version 20, Chicago, IL, USA). We used descriptive statistics to assess the frequency of variables, the chi-squared test, the Fisher’s exact test, or ANOVA for comparing groups. P-values less than 0.05 were considered statistically significant. The patients who had active bleeding or provided inadequate specimens were excluded from the study.

## 3 RESULTS

Sixty-eight patients with anthracotic plaques were included in the study. The patients’ demographic data are presented in Table **1**. The mean ± SD age of the subjects was 72.12 ± 13.74 years. The majority of patients were female (58.8%) and Iranian (85.3%). Most patients had disseminated plaques (86.8%), and obstructive plaques were evident in 48.5% of cases. The numbers of tuberculosis cases diagnosed using various tests are provided in Table **2**. In total, ten patients (14.70%) were reported with positive tuberculosis on BAL smear, BAL culture, tissue biopsy smear, tissue biopsy culture, or by the presence of granuloma in pathologic evaluations. Granuloma lesions were present in 4.41% (3 of 68) and were only seen in Group A samples. In one case of granuloma lesion, microbiological tests of lavage and biopsy samples were negative.

The microbiological test results of biopsy samples for *Mycobacterium tuberculosis* were almost similar comparing Group A and Group B. In all cases, cultures showed a greater diagnostic value compared to smears, and tissue samples (smear or culture), which proved to have a greater diagnostic value compared to BAL fluid (smear or culture).

When biopsy samples from different locations were assessed, fibrosis was more common in Group A than Group B (10.3% *vs*. 1.5%, *p* = 0.03). Acute inflammation was observed in 97.1% of the samples from Group A and 95.6% of Group B. More than 90% of samples from either group indicated mild acute inflammation, with no significant difference in their frequencies. Metaplasia was observed in both groups of samples (14.7% in Group A *vs*. 11.8% in Group B, *p* = 0.68). Dysplasia was not observed in any samples, and malignancy was observed in only one sample of Group A (Table **3**). After adjusting for the presence of obstruction and status of plaques (localized or disseminated), more severe cases of inflammation were seen in patients with bronchial obstruction and disseminated anthracosis. However, there were no significant differences between the two sample groups regarding the markers of the inflammation severity (Tables **S1** and **S2**).

## 4 DISCUSSION

**Fig. (1):**
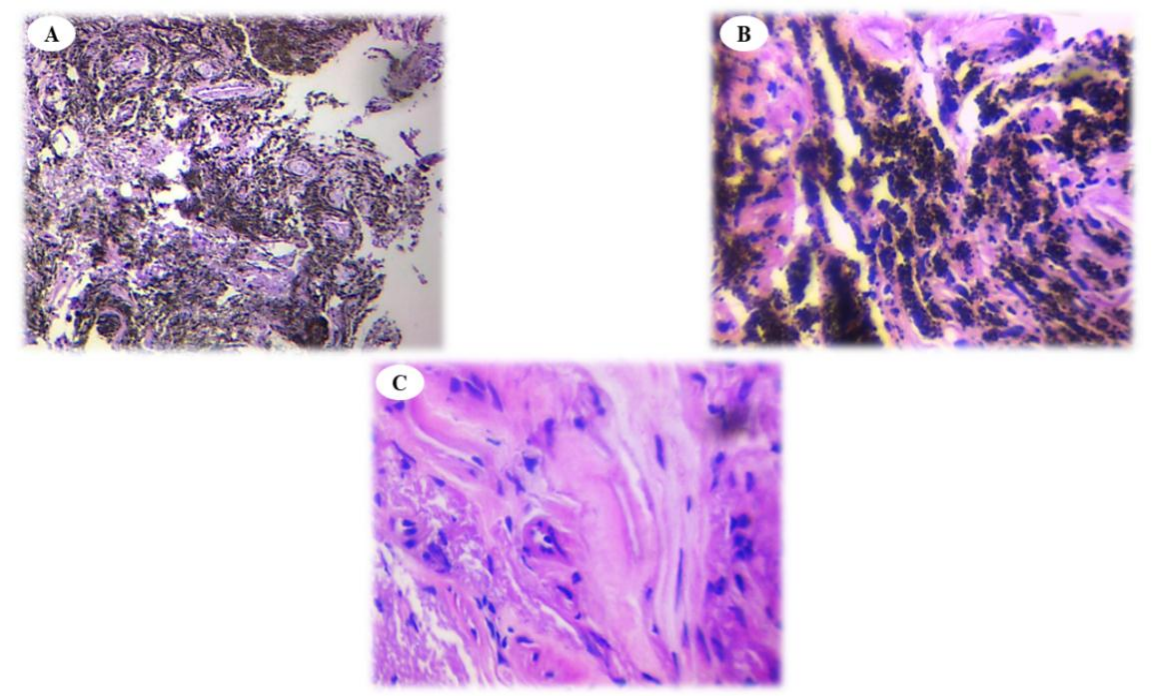
Histologic view of anthracosis-involved tissues (**A**: ×100 and **B**: ×400) compared with a normal periphery (**C**: ×400). Staining method was H&E; Infiltration of inflammatory cells (blue colored cells) is evident for both anthracotic plaques and normal tissue. Black pigmentation is representative for anthracosis.

This study aimed to compare the histopathological features of anthracotic plaques with normal-appearing tissue in patients with anthracosis. We observed that the infiltration of inflammatory cells was similar in anthracotic plaques and normal-appearing tissue (Fig. **1A, B** and **C**). Anthracosis widely involves the lungs and progresses over time. Sandoval *et al*. evaluated 30 patients with pulmonary arterial hypertension and cor-pulmonale who had a long history of exposure to domestic wood smoke. Of them, 22 consented to undergo bronchoscopy, which revealed dark areas in their airways, fibrosis, and acute and chronic inflammation at the basal membrane and submucosa manifesting plasma cell and lymphocyte infiltration [26]. These findings confirm the presence of active inflammatory responses, which were evident in our work in both groups of tissue samples.

Amoli reported ten patients with respiratory symptoms and abnormal respiratory tests with a history of working in a bakery. The patients underwent bronchoscopy and black areas as anthracotic plaques revealed histopathological changes. Infiltration of inflammatory cells such as lymphocytes, plasma cells, and neutrophils was evident in pathologic assessments [27]. Berkheiser examined 1758 autopsies, diagnosed lung anthracosis in 47 patients, and reported that 18 patients with different atypia had metaplasia. The lesions included benign adenomatous proliferation, benign squamous and focal basal cell metaplasia, atypical squamous and focal basal cell metaplasia, and atypical adenomatosis [28]. In the current investigation, metaplasia was observed on both anthracotic and normal-appearing samples in a few cases.

In some studies, researchers suggest a significant association between pulmonary adenocarcinoma and the severity of anthracosis. Patients with anthracotic plaques tend to be in contact with chemicals such as polycyclic aromatic hydrocarbons. It is believed that patients with adenocarcinoma who also have severe anthracosis may have a poor prognosis [29-31]. Amoli studied a group of Iranian patients with pulmonary lesions and found two neoplastic patients who also had anthracosis [15]. Kim *et al*. reported 15 patients (4.9%) with lung cancer and 5 with adenocarcinoma [32]. In our study, only one patient was diagnosed with adenocarcinoma in the anthracotic area, and there is no relationship between malignancy and anthracosis.

Some studies emphasize the increased risk of tuberculosis in patients with bronchial anthracotic plaques [8-11, 13, 14]. We found a 14.7% frequency rate for tuberculosis in the current study. Fekri *et al*. showed a 2.6-fold increased chance of tuberculosis in patients with anthracosis [10]. Pazoki *et al*. found 42 out of 152 (27.6%) bronchial anthracosis patients with pulmonary tuberculosis in their study [13]. Another study reported that 57.8% of anthracotic patients had pulmonary tuberculosis, while only 10.7% of non-anthracotic patients had tuberculosis [11]. In our previous work, 16.4% of patients with anthracosis were also diagnosed with tuberculosis, significantly higher than the control group [14]. Kim *et al*. evaluated 333 patients with bronchial anthracosis plaques and discovered that 113 of them also had active tuberculosis [32]. The rate of tuberculosis is highly different among reports obtained from different cities in Iran and other parts of the world. It is mainly due to the variability in the type of material patients had been exposed to and the degree of exposure to such materials. Furthermore, predisposing risk factors may differ from one part to another part of the country.

Some studies have suggested that aging and inhaling toxic wood smoke are associated with immune system suppression [32, 33]. Mirsadraei *et al*. reported that the prevalence rates of TB in patients with bronchial anthracofibrosis and anthracosis were 22.5% and 32.5%, respectively [12]. We found 14.70% of the anthracotic patients to have TB in the present study, which is similar to our previous study [14]. Chung and colleagues evaluated 28 patients undergoing bronchoscopy and found that 22 of them had obstruction or stenosis, and 17 (60%) had active tuberculosis. Thus, they considered TB infection a contributing factor to fibrosis resulting in stenosis [23]. Our study confirmed this claim by finding a significant association between a positive smear of BAL fluid and obstruction. Kim *et al*. noticed stenosis as one of the most frequent bronchoscopic findings in anthracotic patients: 308 patients (92.5%) had stenosis at least in one part of the bronchial tree, and 232 patients (69.1%) had narrowing at more than one part [32].

Since anthracosis is not a very common condition, much of the literature on this condition is in the form of case reports. In a case report done by Aujayeb *et al*., anthracofibrosis was diagnosed in an 82-year-old woman who had no history of exposure to predisposing factors such as smoking, except for previous involvement with purulent bronchitis and hemoptysis. They reported that TB culture and acid-fast bacilli staining were negative for this patient. Although antituberculosis chemotherapy was started for this patient, paraclinical findings did not confirm TB. However, the patient was well on the follow-up, and no change was observed in paraclinical findings, including the radiological appearance, in addition to clinical status [34]. Similar negative results for TB smear and culture were observed in our study. The negative results from both studies and other reports by other investigators indicate that the main cause of anthracosis may not be an infection. Immunological responses to inflammation due to the deposition of foreign bodies in the respiratory system could be the leading cause of radiologic and histologic findings for anthracosis.

In the current study, in addition to the presence of anthracosis and its extent in the airways, we evaluated the presence and type of inflammatory cells in the bronchial epithelium and histological evidence of malignant cells and *Mycobacterium tuberculosis*. Interpretation of the results indicates that despite the cessation of contact with fossil fuels, the process of inflammation in the bronchi continues. This process is seen at the site of anthracosis plaques and in adjacent areas, perhaps leading to deformities in bronchi and increased risk of infection with *Mycobacterium tuberculosis*. It can also cause metaplasia changes in epithelial cells due to the continuation of this inflammatory process. Therefore, it seems that anti-inflammatory drugs should be considered for these patients before causing irreversible complications such as deformity and obstruction of airways. In addition, periodic clinical visits should be performed, as in any chronic lung disease, and if malignancy or chronic infections such as tuberculosis are suspected, bronchoscopy should be performed.

## CONCLUSION

In conclusion, anthracosis disease seems to cause an inflammatory reaction around the anthracotic plaque and throughout the airways due to the inhalation of dust particles, and this process continues even after exposure cessation. So, Firstly, it is suggested to take a biopsy from seemingly intact tissue near the anthracotic plaque when a biopsy is needed in a patient with anthracosis to reduce the risk of bleeding. Secondly, it seems that anti-inflammatory drugs should be prescribed for these patients before they lead to irreversible complications such as deformity and obstruction of airways.

## Figures and Tables

**Table 1:** Characteristics of patients with anthracosis (n = 68).

**Age, Mean ± SD (Range), y**		72.12 ± 13.74 (23-91)
**Sex frequency (%)**	Female	40 (58.8%)
	Male	28 (41.2%)
**Nationality frequency (%)**	Iranian	58 (85.3%)
	Non-Iranian	10 (14.7%)
**Plaque frequency (%)**	Localized	9 (13.2%)
	Disseminated	59 (86.8%)
**Obstruction frequency (%)**	Yes	33 (48.5%)
	No	35 (51.5%)

**Table 2:** Tuberculosis status in patients with anthracotic plaque (n = 68).

**Diagnostic Tools**	**n (%)**
**BAL Smear**	5 (7.35%)
**BAL culture**	7 (10.3%)
**Bx smear**	5 (7.35%)
**Bx culture**	8 (11.76%)
**Total**	10 (14.70%)

BAL: Bronchoalveolar lavage

**Table 3:** Histopathological comparison of biopsies taken from anthracotic plaques (Group A) and normal-appearing tissue (Group B).

**Pathological Finding**	**Group A (n = 68)**	**Group B (n = 68)**	**P Value**
Fibrosis	7 (10.3%)	1 (1.5%)	**0.03**
Granuloma	3 (4.4%)	0 (0.0%)	NA
Acute inflammation Mild Moderate Severe	66 (97.1%)	65 (95.6%)	0.368
	63 (92.6%)	62 (91.2%)	
	1 (1.5%)	2 (2.9%)	
	2 (2.9%)	1 (1.5%)	
Chronic inflammation Mild Moderate Severe	68 (100%)	65 (96.6%)	NA
	64 (94.1%)	61 (89.7%)	
	2 (2.9%)	3 (4.4%)	
	2 (2.9%)	1 (1.5%)	
Metaplasia	10 (14.7%)	8 (11.8%)	0.687
Dysplasia	0 (0.0%)	0 (0.0%)	NA
Malignancy	1 (1.5%)	0 (0.0%)	NA

## Data Availability

All of the data in excel is available for sharing. Also, the bronchoscopic report and pathologic report is available in Shahid Sadoughi Hospital, Yazd, Iran.
