# Supplementary material for: Inflammation Progresses to Normal Tissue in Patients with Anthracosis after Discontinuation of Exposure to Fossil Fuel
Source: Open Respir Med J. 2022 May 31;16:e187430642203310. doi: 10.2174/18743064-v16-e2203310 (PMC10156033; doi:10.2174/18743064-v16-e2203310)
Supplement: Supplementary file 1 — Supplementary material is available on the publisher’s website. [file TORMJ-16-e187430642203310_SD1.pdf]

1874-3064/22

Send Orders for Reprints to reprints@benthamscience.net

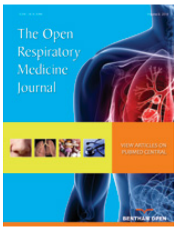

The Open Respiratory Medicine Journal

Content list available at: https://openrespiratorymedicinejournal.com

Supplementary Material

Inflammation Progresses to Normal Tissue in Patients with Anthracosis after Discontinuation of Exposure to Fossil Fuel

Mohammad Samet[1,*], Fariba Binesh[2], Sanaz Zand[3], Mohammad Rezaeisadrabadi[4] and Ryan Nazemian[5]

[1]*Department of Internal Medicine, Shahid Sadoughi Hospital, Shahid Sadoughi University of Medical Sciences, Yazd, Iran*
[2]*Department of Pathology, Shahid Sadoughi Hospital, Shahid Sadoughi University of Medical Sciences, Yazd, Iran*
[3]*School of Medicine, Shahid Sadoughi University of Medical Sciences, Yazd, Iran*
[4]*Department of Internal Medicine, Shahid Beheshti University of Medical Sciences, Tehran, Iran*
[5]*Institute for Transformative Molecular Medicine, Case Western Reserve University, 10900 Euclid Ave, Cleveland, OH 44106, United States*

Table S1. Histopathological comparison of biopsies taken from anthracotic plaques (Group A) and normal appearing tissue (Group B) after adjusting for the presence of obstruction.

	Obstruction	Group A (Anthracotic)			Group B (Normal Appearance)		
		Positive	Negative	P Value	Positive	Negative	P Value
Acute Inflammation	Negative	1	1	0.596	2	1	0.975
	Mild	32	31		31	31	
	Moderate	0	1		1	1	
	Severe	2	0		1	0	
Chronic Inflammation	Negative	0	0	0.482	1	2	0.361
	Mild	32	32		31	30	
	Moderate	1	1		2	1	
	Severe	2	0		1	0	

Table S2. Histopathological comparison of biopsies taken from anthracotic plaques (Group A) and normal-appearing tissue (Group B) after adjusting for the status of plaques (localized or disseminated).

	Plaque Inflammation	Group A (Anthracotic)			Group B (Normal Appearance)		
		Localized	Disseminated	P Value	Localized	Disseminated	P Value
Acute Inflammation	Negative	0	2	0.955	0	3	1.000
	Mild	9	54		9	53	
	Moderate	0	1		0	2	
	Severe	0	2		0	1	
Chronic Inflammation	Negative	0	0	0.641	0	3	0.972
	Mild	9	55		9	52	
	Moderate	0	2		0	3	
	Severe	0	2		0	1	

© 2022 Samet *et al*.

This is an open access article distributed under the terms of the Creative Commons Attribution 4.0 International Public License (CC-BY 4.0), a copy of which is available at: https://creativecommons.org/licenses/by/4.0/legalcode. This license permits unrestricted use, distribution, and reproduction in any medium, provided the original author and source are credited.

DOI: 10.2174/18743064-v16-e2203310, 2022, *16*, e187430642203310
